# The relationship between academic assessment and psychological distress among medical students: a systematic review

**DOI:** 10.1007/s40037-014-0148-6

**Published:** 2014-11-27

**Authors:** Mataroria P. Lyndon, Joanna M. Strom, Hussain M. Alyami, Tzu-Chieh Yu, Nichola C. Wilson, Primal P. Singh, Daniel P. Lemanu, Jill Yielder, Andrew G. Hill

**Affiliations:** 1 South Auckland Clinical Campus, The University of Auckland, Middlemore Hospital, Private Bag 93311, Auckland, Otahuhu 1640 New Zealand; 2 Ko Awatea, Counties Manukau District Health Board, Auckland, New Zealand; 3 Department of Surgery, South Auckland Clinical Campus, The University of Auckland, Auckland, New Zealand; 4 Counties Manukau District Health Board, Auckland, New Zealand; 5 Department of Surgery, The University of Auckland, Auckland, New Zealand; 6 Medical Program Directorate, The University of Auckland, Auckland, New Zealand; 7 Centre for Medical and Health Sciences Education, The University of Auckland, Auckland, New Zealand

**Keywords:** Medical education, Medical students, Curriculum, Academic assessment, Medical student psychological distress

## Abstract

A systematic review was conducted to determine the relationship between academic assessment and medical student psychological distress with the aim of informing assessment practices. A systematic literature search of six electronic databases (Medline, Medline IN PROCESS, PubMed, EMBASE, Psychinfo, ERIC) from 1991 to May 2014 was completed. Articles focusing on academic assessment and its relation to stress or anxiety of medical students were included. From 3,986 potential titles, 82 full-text articles were assessed for eligibility, and 23 studies met review inclusion criteria. Studies focused on assessment stress or anxiety, and assessment performance. Consistent among the studies was the finding that assessment invokes stress or anxiety, perhaps more so for female medical students. A relationship may exist between assessment stress or anxiety and impaired performance. Significant risks of bias were common in study methodologies. There is evidence to suggest academic assessment is associated with psychological distress among medical students. However, differences in the types of measures used by researchers limited our ability to draw conclusions about which methods of assessment invoke greater distress. More rigorous study designs and the use of standardized measures are required. Future research should consider differences in students’ perceived significance of assessments, the psychological effects of constant exposure to assessment, and the role of assessment in preparing students for clinical practice.

## Background

Medical training has been well documented in the literature as a time of high stress and anxiety for many medical students. A systematic review by Dyrbye et al. [1] showed that psychological distress such as stress, anxiety, and burnout among United States and Canadian medical students was consistently higher than the age-matched general population. Factors such as large course workloads and a concern for academic performance have been identified as contributors to this distress [2].

Furthermore, studies have reported on a trend of increasing stress and anxiety for medical students during periods of academic assessment [3, 4]. Often the perceived difficulty of the assessment, and its importance to progression through training, plays a key role [5]. Problems in recalling information, difficulty in memorizing key points, poor study techniques and lack of confidence and time management skills among the students can be additional stressors [6]. Demographic variables such as gender have also been demonstrated to have an effect, with female medical students tending to experience a greater level of assessment stress and anxiety compared with male students [6, 7].

A further concern is that with increased levels of stress or anxiety, academic performance may be impaired [4]. The Yerkes-Dodson law suggests that increasing levels of anxiety or stress can lead to improved performance up to a certain level after which it can then become detrimental, affecting working memory and coping [8].

As there is an increasing emphasis for medical educators to take into account the health and well-being of medical students in the development of medical curricula, we sought to understand how methods of assessment impact on the psychological distress and subsequent performance of medical students with the aim of informing assessment practices.

In this report, we describe our systematic review of the relationship between academic assessment and psychological distress among medical students to address the following questions:

1. How do specific methods of assessment affect medical student stress or anxiety (the reviewers used these terms to conceptualize psychological distress)?
2. How have researchers measured assessment stress or anxiety (including biological and psychological measures)?
3. Which specific methods of assessment are associated with greater medical student stress or anxiety?
4. What is the relationship between assessment stress or anxiety and performance?
5. What are the limitations of current research and important areas for future investigation?

Due to heterogeneity between the studies, such as the focus on various methods of assessment and use of different instruments to measure stress or anxiety, it was not possible to collate the data for a meta-analysis. Therefore, the reviewers consolidated the studies into a qualitative report with appropriate critical review.

## Methods

To ensure uniformity and clarity, the following definitions were chosen by the reviewers for the purpose of this review. Medical students were defined as students enrolled in tertiary programmes who will eventually qualify as medical doctors. Assessments were defined as specific events that test the knowledge or performance of a medical student.

A literature search of six electronic databases: Medline, Medline IN PROCESS, PubMed, EMBASE, Psych info and ERIC was completed. Key search terms used were in combinations of: examination, or exam, or assessment, or appraisal, or test, or viva voce, or education measurement; and medical student; and medical school or medical curriculum or medical programme; and anxiety or stress or distress or burnout or pressure or coping or concern or worry or apprehension or nervousness or fear or addictive behaviour or depression or psychological disturbance. The search was limited to articles published in English between January 1991 and May 2014. All original research articles, reviews, editorials, and essays were retrieved for examination and a bibliography management programme (ENDNOTE X3, Thomson Reuters, New York) was used to create a search library. The search yielded a total of 2,781 hits (after removal of duplicates). An additional 16 papers from a hand search of key journals and from reference lists were included.

The reviewers met to determine the review inclusion and exclusion criteria, and to select articles for critical appraisal and review. These criteria are listed below:

## Inclusion criteria

1. All study participants are medical students.
2. Description of specific features of an assessment.
3. Measured level of stress/well-being.
4. Study focuses on the effect of assessment/examination/evaluation on the anxiety/stress/distress/burnout/pressure/coping/concern/apprehension/fear/addictive behaviour/depression/psychological distress of medical students.

## Exclusion criteria

1. Full-text of article not published in English.
2. Studies published prior to 1990 (as we wanted to focus on current assessments used in medical education with contemporary measures of stress and anxiety).
3. Study results duplicated in separate earlier publications.
4. Brief descriptive, commentary or review article.

**Fig. 1:**
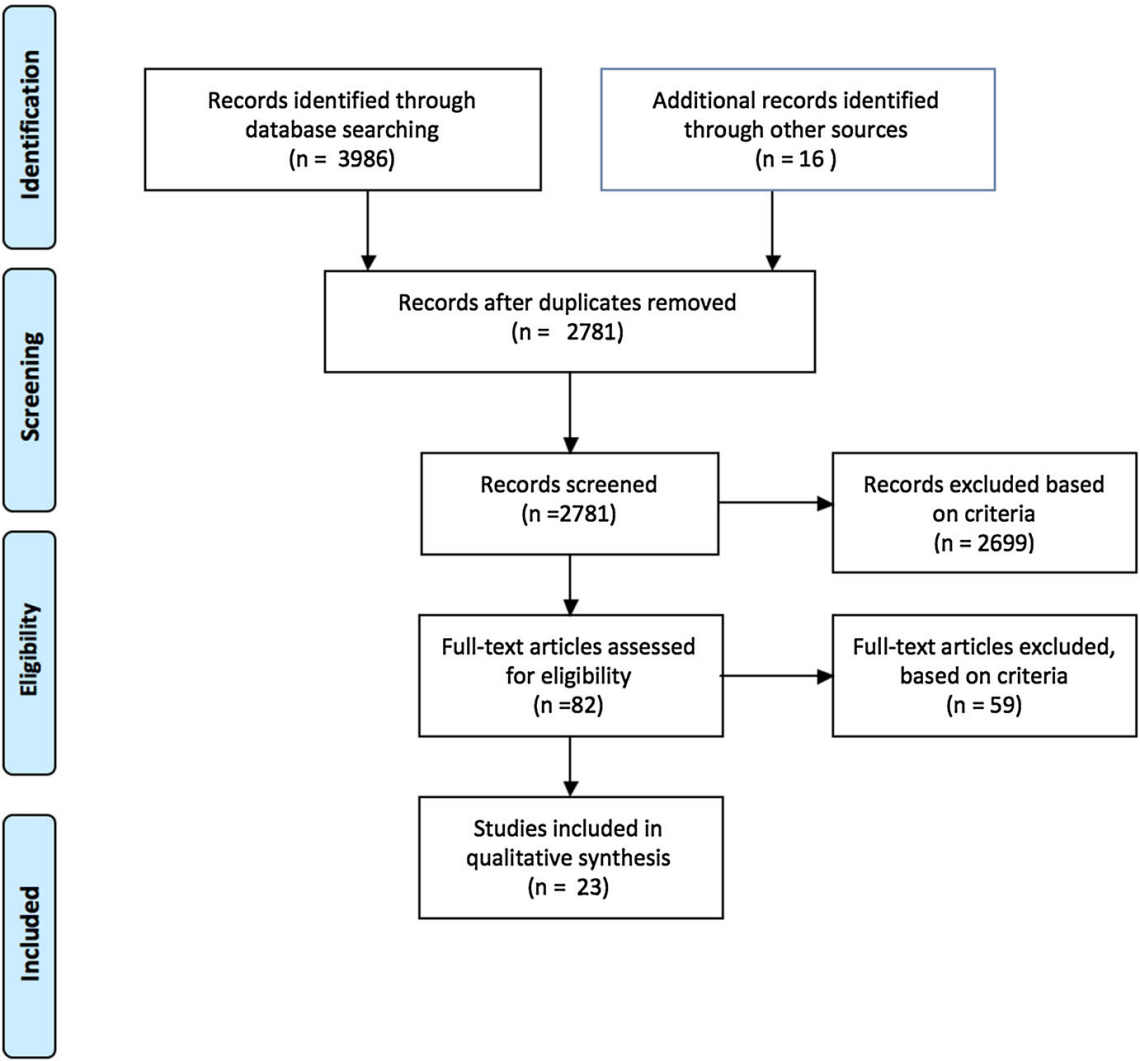
PRISMA statement of search results [46]

In August 2011, a total of 100 articles were identified by two independent reviewers [Author 2, Author 4] as potentially relevant studies after screening the titles and online abstracts of the initial 1,813. From the 100 articles identified, the inclusion and exclusion criteria were used to select 57. These were obtained as full texts for examination. The selection of papers for this review was completed by October 2011 and a total of 11 studies were selected. Following a further literature search in May 2014, an additional 968 studies were identified (excluding duplicates). After screening titles and online abstracts, the inclusion and exclusion criteria were used to identify 24 full-text papers for review, and a total of 12 studies were selected (Fig. 1).

**Fig. 2:**
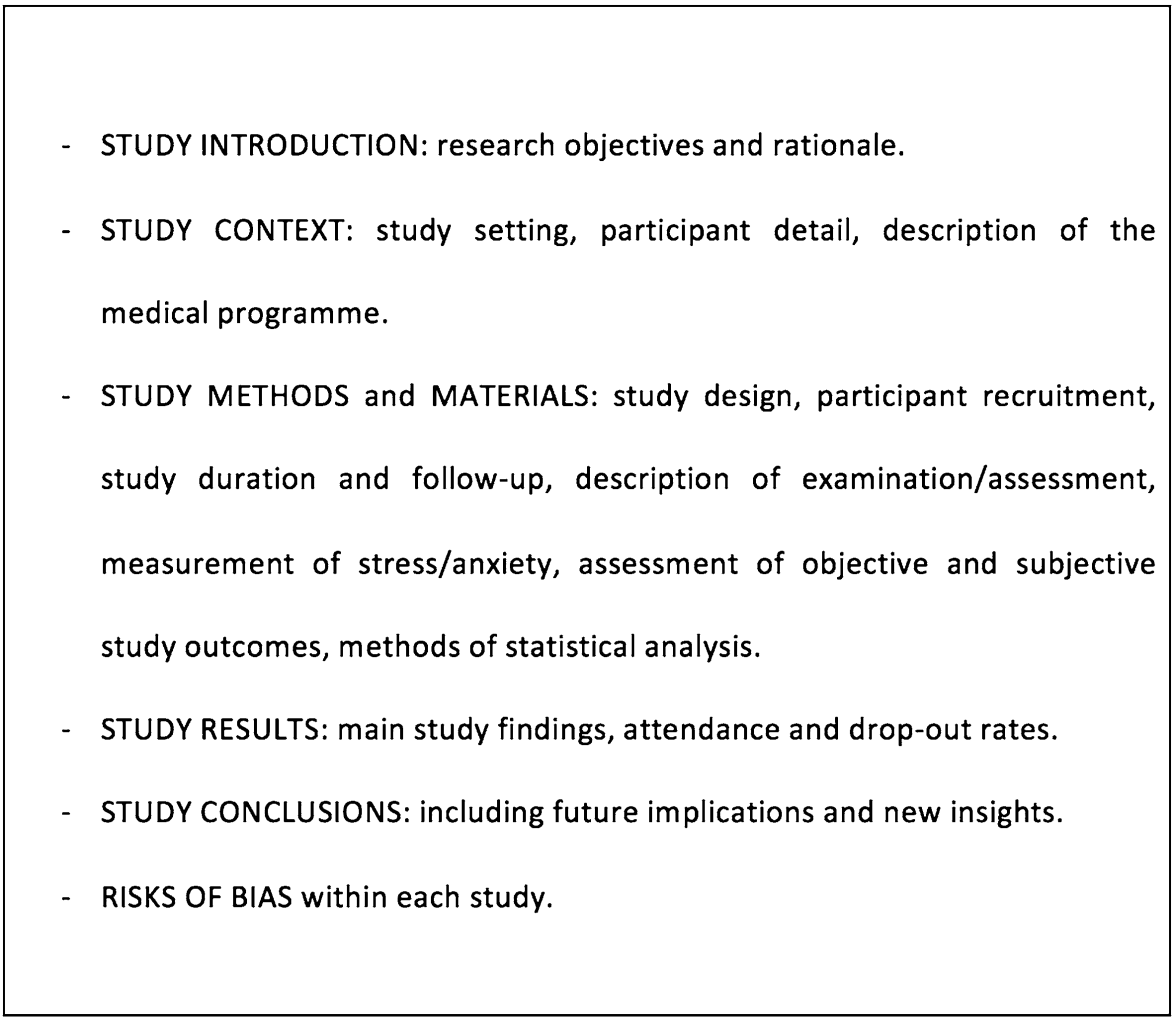
Data extraction and critical appraisal framework

Each selected article was analyzed independently by at least two reviewers and data extracted using a critical appraisal framework (Fig. 2). To assess study quality, the reviewers considered a variety of factors that could contribute to risks of bias within each study including recruitment methods, outcomes assessment, and reporting of outcomes. The data extraction and review instrument also asked reviewers to draw conclusions about the validity and reliability of the evidence presented in each article. Any discrepancies in opinion between the reviewers were identified and final agreement was reached after face-to-face discussion.

## Results

This systematic review included 23 studies that fulfilled the inclusion criteria [6, 7, 9–29].

## Study settings

A total of seven studies were set in the United States originating from different medical faculties [9–15]. Five studies were from India, the next most common setting [16–20]. Studies were conducted in dedicated medical programmes. The majority of studies did not provide details of those programmes.

## Study participants

The year level of participating medical students ranged from Year 1 to Year 5 (final year). One study included premedical students in addition to medical students as study participants [9].

Demographic information including the age and gender of participants was reported by 14 studies [6, 7, 10, 12, 15–24]. A further six studies reported gender but no comment was made on the age of participants [11, 14, 25–28]. Three studies did not report demographic variables [9, 29].

Assessment of baseline levels of participants’ perceived stress or anxiety was completed in nine studies [10, 17, 18, 21–23, 25, 26, 29]. Seven studies assessed participants’ baseline levels of stress using physiological parameters such as blood pressure or heart rate [9, 10, 16, 17, 19, 20, 23].

## Study outcome measures

There were a variety of subjective outcomes measured in the studies. Self-reported survey questionnaires were the most common form of evaluation created or adopted by investigators to evaluate the outcome measures. Investigators generally provided a brief description of the questionnaire. Six studies utilized Spielberger’s Test Attitude Inventory (TAI) [7, 12, 21, 23, 28, 29]. This Inventory is a self-reported psychometric scale used to measure individual differences in test anxiety [18]. Post-assessment interviews were a further subjective measure in one study [14].

Investigators also measured a range of objective physiological measures of stress or anxiety. For example, one study measured pulse, blood pressure and carried out an electrocardiogram (ECG) on participants at the time of a viva voce assessment [19]. Another study measured the auditory reaction time, eosinophil count and galvanic skin resistance prior to a viva voce assessment [20]. Three studies used salivary or blood biochemical markers as correlates for anxiety or stress levels which included cortisol, thyroid profiles, and MiRNA levels [10, 17, 21].

## Study results

Studies addressed the effect of specific methods of assessment on participant stress and anxiety. Broyles et al. [14] evaluated whether an open-book setting for assessment reduces stress during a family medicine clerkship. During the study period, two sites used a closed-book setting for final written assessments in 2001. In 2002, the experimental site used an open-book setting for the final written assessment. Participants were interviewed immediately following this assessment. Answers were analyzed using qualitative methodologies and where indicated, percentages of responses were calculated within a theme. Key themes were generated to answer the study question. Over 80 % of the participants described feelings of being less stressed about being assessed in an open-book setting.

Reteguiz [12] utilized the TAI to assess levels of anxiety following a standardized patient assessment (SP) and a multiple choice question (MCQ) assessment during an internal medicine clerkship. Participants were categorized with low, moderate, or high levels of anxiety. Study findings suggested a moderate level of anxiety associated with both SP and MCQ assessment methods. However, there were no significant differences in levels of anxiety between the assessments.

Anxiety or stress associated with clinical assessments was investigated in seven studies [11, 12, 16, 19, 20, 24, 29]. The majority of study participants experienced increased levels of anxiety or stress associated with this type of assessment. This was demonstrated by elevated stress and anxiety scores reported from survey questionnaires, or an increase in physiological measures such as pulse rate and blood pressure at the time of the assessment.

## Grading systems and psychological distress

A single study investigated the effect of grading systems on medical student psychological distress. Bloodgood et al. [15] compared the impact of a change from a graded (A, B, C, D) to a pass/fail grading system on medical student anxiety, depression, positive well-being, self-control, vitality, and general health. Two cohorts of medical students were surveyed, the last cohort under the five-interval grading system and the first cohort under the pass/fail grading system. The medical curriculum was reported as essentially identical between cohorts. Well-being was self-assessed by students with a web survey utilizing the Dupuy Schedule of General Well-Being. The pass/fail cohort exhibited a statistically significant increase in measures of well-being (*P* < 0.01) compared with the graded cohort.

The effect of an ‘honours’ system on levels of stress within the pass/fail class was also assessed in the same study. Within the pass/fail cohort the top 20 % of students were awarded ‘honours’. In a separate survey within this study, 70 % of students who consciously chose to seek honours felt that this resulted in greater stress. In comparison, 92 % of students who consciously chose to not pursue honours reported this decision resulted in lower stress.

## Psychological distress and assessment performance

Three studies addressed the influence of stress or anxiety on assessment performance. Reteguiz [12] found no evidence of an inverse relationship between anxiety level and performance as measured by the TAI, and SP and MCQ assessment scores suggesting performance was not impaired by anxiety. This finding conflicts with evidence reported by Frierson et al. [13] which indicated a relationship exists between anxiety and impaired performance on the National Board of Examiners examination. Similarly, Farooqi [7] reported an inverse relationship between test anxiety and academic performance. Yusoff [26] also reported a relationship between moderate to high stress levels, and higher risk of failing an examination.

Hettiarachchi and colleagues [25] demonstrated a relationship between quality of life (QoL), and assessment performance. They found students who performed better at an examination had significantly higher QoL scores at each domain tested (physical, psychological, social, and environmental QoL). In contrast, students with lower QoL scores tended to perform more poorly.

A significant gender effect on anxiety and performance was suggested by Pamphlet et al. [28] with lower anxiety being positively associated with higher performance of female medical students in an MCQ assessment. However, this finding was not consistent with a study by Reteguiz, which found no gender differences in relation to assessment anxiety and performance [12].

## Factors associated with assessment stress and anxiety

An interaction between gender, and assessment stress or anxiety was detected within many of the studies. Higher levels of assessment stress or anxiety were found among female medical students [6, 7, 11, 16, 24, 30]. However, many studies in this review also found no relationship between gender and assessment stress or anxiety [10, 22, 23, 26].

Hashmat et al. [24] observed several factors that contributed to assessment anxiety among final year medical students. Extensive course work load, the long duration of periods of assessment, lack of assessment preparation and a lack of exercise were reported to be the most important contributing factors. Many students also had little knowledge of assessment-taking and anxiety-reduction techniques. Yusoff [26] identified additional examination stressors, which included large amounts of content to be learnt for an examination, lack of time to review what had been previously learnt, self-expectation to perform well, and concern about getting poor marks.

Ping et al. [29] evaluated the pattern of anxiety symptoms during the course of a clinical assessment. Anticipatory anxiety was found to be the most prominent form of anxiety experienced with symptoms peaking 10 min before an assessment. These symptoms quickly subsided as the assessment proceeded. No resurgence of anxiety symptoms was reported in the presence of examiners. Students with high-test anxiety trait scores as measured by the TAI had significantly more anxiety symptoms during the assessment in comparison with students with lower scores.

## Methodological quality of studies

### Study design

All 23 studies identified were observational studies with most using an observational cohort study design. Seven studies used a comparison group for assessment of outcomes [6, 10, 11, 14, 15, 18, 26]. In general, descriptions of study design by authors were limited preventing replication of study methods. Study sample sizes varied significantly; ranging between 10 and 450 participants. Variable response rates were also reported between studies (52–98 %).

### Participant sampling and selection bias

Participant recruitment or sampling methods were not described in the majority of studies in this review. Of the remaining studies discussing recruitment, two studies observed entire class cohorts creating a potential for unmatched differences between cohorts [11, 15]. Participation in these studies was based on voluntary self-selection. Only one study noted any explicit inclusion/exclusion selection criteria for participants [25].

### Outcome assessment bias

In relation to reliability of assessment methods, one study discussed reliability of the SP and MCQ assessment, which was reported as 0.63 and 0.80 respectively [12]. Colbert-Getz et al. [11] also reported on the reliability of the SP assessment used in their study (Cronbach alpha was 0.64) [12]. No other studies commented on assessment reliability.

The psychometric properties of the outcome evaluation tools employed were seldom reported. However, six studies reported the use of the TAI to measure participant anxiety levels [12, 21–23, 28, 29]. This Inventory is considered a valid and reliable instrument of test anxiety [12, 31]. Four studies reported the use of other measures of stress or anxiety including the Dupuy Schedule of General Well-Being, Zung’s Scale, an emotionality-worry scale, and a six-point Likert scale measuring anxiety [11, 13, 15, 16]. According to Bloodgood [15], whilst the Dupuy Schedule of General Well-Being is well validated in clinical studies, it has not been previously used in undergraduate medical education. Internal consistency of the emotionality-worry scale was reported (0.81 and 0.86 for each of the subscales) [13]. Discussion of the validity of Zung’s Scale was not reported. As Colbert-Getz et al. [11] measured student test anxiety with one survey item containing a six-point Likert scale, they could not determine the psychometric properties of anxiety ratings in their study.

Hettiarachchi et al. [25] chose to use the World Health Organization Quality of Life Questionnaire (WHOQOL-BREF) instrument which has been documented for its reliability and validity [32]. Furthermore, the psychometric properties of the WHOQOL-BREF when applied to medical students has previously demonstrated adequate internal consistency and reliability [33].

A number of studies utilized physiological measures such as heart rate, blood pressure, and cortisol levels, as a measure of stress or anxiety. However, changes in cortisol levels are not necessarily specific to stress or anxiety [34]. Additionally, Zeidner [35] considers physiological measures of assessment anxiety to be problematic in both a practical sense and in terms of construct validity.

Two studies examined alterations in miRNA profiles of medical students in order to measure stress responses prior to, and following an assessment [21, 22]. These alterations were then correlated with psychological anxiety as measured by the TAI. Although study authors hypothesize an elevated miR-16/miR-144r may be a stress response to assessment-related stress and anxiety, the authors conclude further studies are needed to prove their hypothesis.

A possible response bias was present in a study by Ping et al. [29], which did not measure actual assessment performance but rather only perceived performance as reported by study participants. As a result a social desirability bias may have occurred. This refers to an individual not adhering to a social norm but reports a socially desirable behaviour when questioned [36]. Study authors did not consider the potential impact of this bias on the validity of their research.

### Reporting bias

Incomplete data collection and selective outcome reporting were potential sources of bias in all reviewed studies. Reasons for incomplete or missing data were commonly not described. The variable response rate between studies and the significance of non-participation bias were not addressed in the reviewed studies. In contrast with these sources of bias, most studies were at low risk of selective outcome reporting as primary outcomes were measured and reported.

## Discussion

This systematic review identified 23 studies that evaluated the relationship between academic assessment and psychological distress among medical students. Studies included a variety of assessment methods ranging from open or closed-book assessments, to MCQ, SP, OSCE and viva voce. A variety of methodologies including psychological and physiological measures were used to determine the impact of these forms of assessment on participant stress and anxiety. As the studies varied significantly, the degree of heterogeneity between these studies meant direct comparison was not possible. However, consistent among the studies was the finding that assessment invokes stress or anxiety, perhaps more so for female medical students. However, a gender effect was not consistent across all studies included in this review, and therefore its significance remains unclear.

A variety of factors can contribute to assessment stress and anxiety among medical students. Psychological factors are considered a leading cause of assessment anxiety [24]. Zeidner [35] suggested that students experiencing assessment anxiety are shown to be preoccupied with negative self-referential thoughts including doubts about academic competence and fear of failure. Also of significance is the contribution of course load, duration of the assessment period, and lack of knowledge of assessment and anxiety-reduction techniques [24]. These findings suggest that in addition to psychological factors, the learning environment may also contribute to assessment anxiety.

A relationship may exist between assessment stress or anxiety and assessment performance, although, this finding was not consistent across all studies included in this review. Therefore, the Yerkes-Dodson law could not be substantiated when applied in the context of medical education and assessment practices. The Yerkes-Dodson law has been cited in this review because it is frequently mentioned in the literature on personal stress [37]. However, it has limitations when used as a model as it does not allow for causes, perceptions, or coping mechanisms for stress or anxiety [37]. Furthermore, as it is not specific to stress or anxiety, but rather a theory of physiological arousal; more appropriate frameworks for this relationship could be stress specific theories such as the ‘Theory of Cognitive Appraisal’ by Lazarus and Folkman [38]. This theory can elaborate further on the relationship between psychological distress and assessment performance, by considering both the meaning and potential consequences of assessments for students, and the associated coping mechanisms. The stress of an assessment could relate to its perceived value, for example, whether an assessment is formative or summative, or its importance to progression through medical school. The potential consequences of this distress may be the development of adaptive or maladaptive coping mechanisms. Students may proactively cope by optimizing study techniques and time management, or by seeking social support and additional tutoring; or they may cope maladaptively by adopting avoidant behaviours, mentally disengaging, or by alcohol and drug abuse [39].

The limitations of this review are derived mainly from limited reporting of study procedures or methodologies, which limited the extent to which reviewers could draw firm conclusions about assessment stress or anxiety. These limitations also extend to risks of bias throughout the reviewed studies. Self-selection bias was likely present among most studies in this review. This was heightened by a lack of description of participant selection methods and low participant response rates in many of the studies. A gender bias may also exist as a result of self-selection. The significance of social desirability bias must also be considered a result of self-reporting within many of the reviewed studies. Social desirability bias affects the validity of a questionnaire and can confound relationships between variables by obscuring or producing artificial relationships among the variables [36]. The majority of the articles reviewed provided only a brief outline of the questionnaires used. Researchers using questionnaires should consider the effect of socially desirable responding on the validity of their research.

This review has implications for assessment practices for medical students. Faculty should consider the impact of assessments on student stress and anxiety, particularly as future clinical practice is inherently stressful [40, 41]. As students enter the workforce, the ability of students to cope may be eroded by their undergraduate training [42, 43]. A counter argument is that the coping skills and resilience required for the workforce may be developed through experiences such as assessment. Faculty could also play a role in preparing students to cope with the stress associated with assessment [44]. Interventions such as counselling services and stress management programmes are frequently reported in the literature as having a positive effect [24, 45].

An important gap in the literature is a lack of evidence that compares potential differences between high-stake or low-stake assessments, and formative or summative testing, on medical student stress and anxiety. Furthermore, the research currently available is limited by short follow-up durations and does not include information on the effect of ongoing exposure to assessment over the long-term.

## Conclusions

There is evidence to suggest academic assessment is associated with psychological distress among medical students. However, differences in the types of measures used by researchers limited our ability to draw conclusions about which methods of assessment invoke greater distress. More rigorous study designs and the use of standardized measures are required. Future research should consider differences in students’ perceived significance of assessments, the psychological effects of constant exposure to assessment, and the role of assessment in preparing students for clinical practice.

## Essentials

- Assessment invokes stress and anxiety, perhaps more so for female medical students.
- Assessment stress and anxiety can impact performance.
- Grading systems and assessment methods can increase student stress and anxiety.
